# Genomics Associated Interventions for Heat Stress Tolerance in Cool Season Adapted Grain Legumes

**DOI:** 10.3390/ijms23010399

**Published:** 2021-12-30

**Authors:** Jitendra Kumar, Reyazul Rouf Mir, Safoora Shafi, Debjyoti Sen Gupta, Ivica Djalovic, Jegor Miladinovic, Rahul Kumar, Sachin Kumar, Rajeev Kumar

**Affiliations:** 1 Division of Crop Improvement, ICAR-Indian Institute of Pulses Research, Kanpur 208024, India; debgpb@gmail.com; 2 Division of Genetics and Plant Breeding, Faculty of Agriculture (FoA), Wadura Campus, SKUAST-Kashmir, Sopore 193201, India; safoorashafi999@gmail.com; 3 Institute of Field and Vegetable Crops, National Institute of the Republic of Serbia, Maxim Gorki 30, 21000 Novi Sad, Serbia; jegor.miladinovic@ifvcns.ns.ac.rs; 4 Department of Genetics and Plant Breeding, Ch Charan Singh University, Meerut 250005, India; rkdsum@gmail.com (R.K.); sachinkpsingh@gmail.com (S.K.); 5 Faculty of Agriculture, C.S.S.S.P.G College, Meerut 250106, India; rnain1972@gmail.com

**Keywords:** climate change, high temperature, epigenetics, genome editing, nanoparticles, candidate genes, mRNA, signalling pathways

## Abstract

Cool season grain legumes occupy an important place among the agricultural crops and essentially provide multiple benefits including food supply, nutrition security, soil fertility improvement and revenue for farmers all over the world. However, owing to climate change, the average temperature is steadily rising, which negatively affects crop performance and limits their yield. Terminal heat stress that mainly occurred during grain development phases severely harms grain quality and weight in legumes adapted to the cool season, such as lentils, faba beans, chickpeas, field peas, etc. Although, traditional breeding approaches with advanced screening procedures have been employed to identify heat tolerant legume cultivars. Unfortunately, traditional breeding pipelines alone are no longer enough to meet global demands. Genomics-assisted interventions including new-generation sequencing technologies and genotyping platforms have facilitated the development of high-resolution molecular maps, QTL/gene discovery and marker-assisted introgression, thereby improving the efficiency in legumes breeding to develop stress-resilient varieties. Based on the current scenario, we attempted to review the intervention of genomics to decipher different components of tolerance to heat stress and future possibilities of using newly developed genomics-based interventions in cool season adapted grain legumes.

## 1. Introduction

Cool season grain legumes are rich in proteins, vitamins, and minerals such as iron, zinc, and folate. Hence, their intake in daily diet provides solution of overcoming the problem of malnutrition and mineral deficiencies among the poor people of developing countries who cannot afford costly animal protein-based diets. Moreover, use of grain legumes provides a remedy for several chronic diseases like diabetes, obesity, and cardiovascular problems [1]. Therefore, health conscious people now prefer use of plant-based protein in their diets even in developing countries over animal-based proteins [2]. This is resulted in increasing the demand of grain legumes day by day. However, several biotic (i.e., wilt, rust, blight diseases) and abiotic (i.e., heat, drought, salinity, acidity and water logging) stresses significantly affect the yield potential of current cultivars of food legumes [2,3]. Among abiotic stresses, heat stress is increasingly becoming a serious problem for the production of cool season grain legumes due to climate changes [4]. Heat shock and moderate heat stress are two types of heat stress. First is occurred due to lethal temperatures for a short period of time, while later one is commenced when temperatures arise above the optimum level for a long period of time [5]. Moderate heat stress generally affects the growth and development of cool season legumes. In India, a large shift in area of grain legumes from cooler, long season environments to warm, short season environments has been made in the past years due to changes in the environmental conditions. For example, the area under chickpea cultivation has been shifted from northern to southern India. Many other countries also could experience unprecedented heat stress due to global changes in climate. Cool season grain legumes including chickpea (*Cicer arietinum* L.), lentil (*Lens culinaris* Medik.) and faba bean (*Vicia faba* L.) have yield losses if day temperatures are increased from critical day temperatures of 35 °C. Heat stress during flowering reduced yield in pea (*Pisum sativum* L.) [6]. In the common bean (*Phaseolus* spp.), high night temperature (>20 °C) causes yield losses. In India, it is grown as a cool season crop during winter season despite its being a warm season crop.

Heat stress in a complex trait and a network of genes, which controls the physiological and agronomic traits, is involved in tolerance to heat stress. For example, in common bean, enhanced leaf cooling identified as a pathway for heat stress tolerance [7]. Thus, conventional breeding approaches could not be very successful in developing the heat-tolerant cultivars in food legumes due to complex inheritance, except a few cases in chickpea and faba bean [8,9,10] and other warm season crops like cowpea [11]. However, heat-tolerant genotypes have been identified in a number of cool season grain legumes for further utilization in conventional breeding for developing heat-tolerant cultivars [12,13,14,15]. Efforts have been made to improve the heat tolerance through conventional breeding approaches, and screening methodologies have been developed to identify heat tolerant cultivars [15,16]. In recent years, genomics has emerged as a way to decipher the genetics underlying complex traits imparting heat stress tolerance in food legumes and published several reviews focussed on different aspects of heat stress including seed setting [17], functional mechanism [18], heat stress during reproductive and grain-filling phases [19], functional genomics [20], physiological and molecular approach [21], and breeding, genetics and genomics [2]. In the recent past years, new knowledge have been generated in the area of genomics for tackling heat stress tolerance in cool season grain legumes, which were not covered in previously published review articles. Therefore, in this review, we discussed current and future genomics inventions for heat stress tolerance in the context of cool season grain legumes.

## 2. Advances in Screening Techniques for Heat Tolerance

Heat-tolerant genotype has minimum yield losses, when temperature goes beyond the threshold temperature. In cool season grain legumes, critical threshold temperature is varied from 28 to 35 °C [19]. Temperature above 28 °C during reproductive period causes sensitivity of pea crop [22]. While in lentil and chickpea, temperature greater than 35 °C during flowering and podding resulted in poor grain yield [15]. Significant yield losses have been observed in faba bean under daily temperatures > 25 °C [23,24], and stops flowering and produces a few extra leaf-bearing nodes at 30 °C [25]. The critical temperature for heat tolerance seems to be higher in chickpea than faba bean, lentil, and field pea, and the reverse is true for cold tolerance [26]. Thus, this crop shows high sensitivity to heat stress. Heat stress affects several phonological, biochemical and physiological traits such as limited growth rate, membrane instability, photosynthesis, reproductive development, and reduced net assimilation rate [17,24,25,26,27,28,29,30]. Heat stress sensitivity has been observed more in cool season grain legumes compared to warm season food legumes [8].

**Figure 1:**
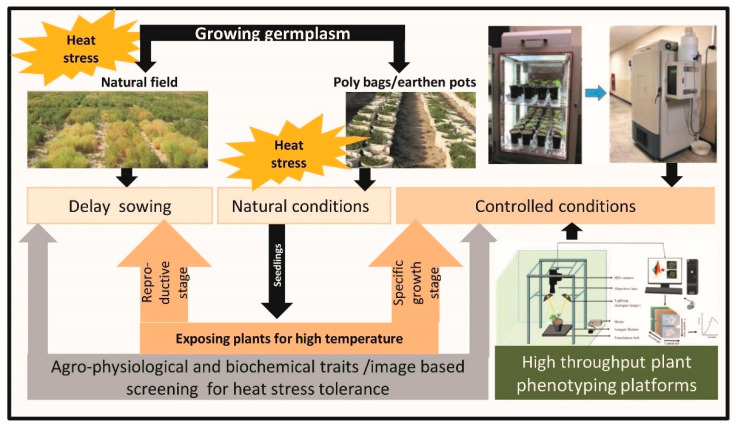
An overview of screening methodologies used for identification of differentiating heat tolerant and sensitive genotypes in cool season grain legumes (modified from [31]).

Different approaches have been used to differentiate the heat tolerant and sensitive genotypes by screening a number of genotypes at the temperature higher than the threshold level in cool season grain legumes (Figure 1).

Delay sowing of genotypes under natural conditions in the field has been used as one of the approaches for this purpose. It is planned in this way that reproductive period could coincide with high temperature (i.e., greater than the threshold temperature). It is widely used in several cool season grain legumes including chickpea and lentil [15]. However, in another study, lentil genotypes were grown in earthen pots following delay sowing in order to ensure heat stress (>30/20 °C; average max./min. temperatures) during seed filling [28]. Advancement in screening technologies resulted in development of several approaches for screening heat stress tolerance in crop plants including cool season grain legumes. These approaches used both natural and controlled conditions to grow plants under heat stress. For this, plants initially were grown under field conditions in earthen pots or poly bags and after that they were transferred in controlled conditions for exposing them to high temperature. Another way used for screening heat stress tolerance is to grow plants of different genotypes in pots under controlled conditions for their whole life cycle and then higher temperature is given at desired stage of plants. In chickpea, polybags have been used to grow different genotypes and 14-days-old seedlings were subsequently exposed to elevated temperature by creating polythene structure [32]. As we know, flowering time and duration are important traits for managing stress through escape. Therefore, efforts have been made to develop the image-based techniques for high-throughput phenotyping of flowering intensity in cool season grain legumes [33]. This study used multiple imaging sensors, image resolution, and image processing techniques in monitoring flowering intensity in chickpea and pea and found strong correlation of image data with visual rating scores in pea (*r* = 0.72). This study demonstrated possibility of using imaging for phenotyping of flowering in crop plants [33]. Besides high-throughput phenotyping of individual plants, advances in phenomics resulted in development of smartphone app platform for field phenotyping at organ level using images and pictures [34]. It is very comfortable to take 2D pictures of targeted organ like leaf angel and leaf length through this. Multi-view stereo (MVS) approach is another way of organ phenotyping at a low cost [35]. Further automation and robotics, new sensors, and imaging technologies (and software) are emerging opportunities for developing high-throughput plant phenotyping platforms (HTPPs) for screening high temperature tolerant genotypes in coming years [36]. Further, technological advances in digital cameras, infrared thermal imagers, light detection and ranging (LIDAR), multispectral cameras, and hyperspectral sensors are becoming helpful in developing of ground-based platforms and aerial-based platforms for phenotyping under field environments [37]. For example, in sorghum, the tractor-based proximal crop-sensing platform, and UAV (unmanned aerial vehicle)-based platform helped to phenotype complex traits like growth and radiation use efficiency (RUE) in sorghum [38].

## 3. Genomics Interventions

**Figure 2:**
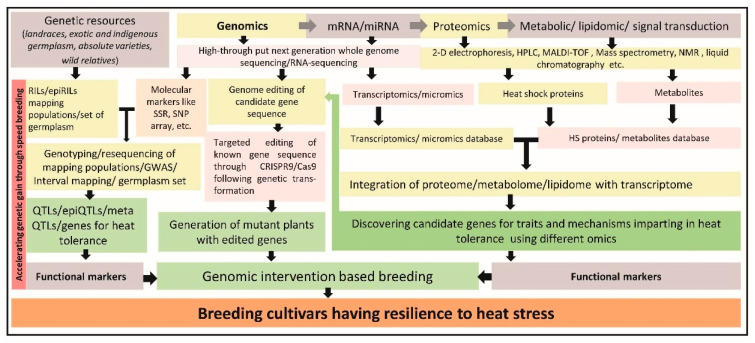
An overview of genomic interventions based breeding strategies for heat stress tolerance in cool season legumes.

In cool season grain legumes, significant progress has been made to enrich genomic resources (Table 1), which helped to develop the different types of molecular markers including SSRs, diversity arrays technology (DArT) markers, single nucleotide polymorphism (SNP) markers, different SNP platforms, micro-array based markers, NGS-based markers, genotyping by sequencing (GBS), InDel markers etc. [39,40,41,42,43] and other genomic-based tools and techniques. Advances in genomics have led to intervention in its use for enhancing the knowledge on heat-stress tolerance through the following ways (Figure 2).

### 3.1. QTL/Gene Mapped for Traits Imparting Heat Stress

Heat stress is a complex trait and many genes control those complex traits that impart heat-stress tolerance. Developments in genomics have made it feasible to dissect genetic architecture of underlying such complex traits through QTL/gene mapping. Molecular markers help to tag the genomic regions (i.e., quantitative trait loci; QTLs) controlling the phenotype of a complex trait, which are distributed throughout the genome.

Each QTL is comprised of many genes, which are investigated as potential candidate genes for a trait under study. Mapping populations derived from two parents (i.e., biparental QTL mapping) and multi-parents/association panel (i.e., association mapping) are usually used to map the QTLs/genes controlling a trait of interest [105,106]. However, markers linked a QTL controlling complex traits have not been used in marker assisted breeding program due to poor markers density within QTL regions. However, next-generation sequencing-based approaches especially genotyping by sequencing (GBS) have provided a large number of evenly distributed SNP and gene SSR markers over genome [107,108]. This led to development of high resolution linkage maps in cool season grain legume crops [109,110,111,112], and are used to map several traits including traits imparting in heat tolerance (Table 2).

In chickpea, a linkage map spanning 529.11 cM and comprising 271 genotyping by sequencing (GBS) based single nucleotide polymorphism (SNP) markers identified major QTL for number of filled pods per plot, total number of seeds per plot, grain yield per plot and % pod setting under heat stress [113]. In some cases, candidate genes controlling associated quantitative traits have been identified [117]. Moreover, genome-wide association studies (GWAS) allow narrowing down the candidate regions to explore specific haplotypes in natural populations and even wild species [118,119]. In chickpea, a recent GWAS was conducted in a panel of 300 accessions to investigate the marker–trait association for heat tolerance [120]. In pea, genome-wide association (GWA) analysis with 16,877 known high-quality SNPs identified association of genomic regions with chlorophyll concentration (6 QTLs), with photochemical reflectance index and canopy temperature (2 QTLs); reproductive stem length (7 QTLs), internode length (6 QTLs) and pod number (9 QTLs) and also identified 48 candidate genes responsible for these traits under heat stress [116]. QTLs for heat tolerance have also been mapped using GWAS in cereals like wheat (*Triticum aestivum* L.) [121,122,123,124,125,126] and jowar or sorghum (*Sorghum bicolor* L.) [127]. Several QTLs for heat tolerance at flowering stage have been identified and used in breeding program for developing heat tolerant cultivars of rice (*Oryza sativa* L.), [128,129,130]. In maize (*Zea mays* L.), QTL hot spots for grain yield under heat stress have been identified [131].

Among food legumes, two dominant genes for heat stress tolerance [132] and QTLs for pod set number per peduncle under HS have been identified in cowpea (*Vigna unguiculata* L.) [133,134]. Further comparative genomic analysis identified HSPs and HSFs in these QTL regions in soybean (*Glycine max* L.) [3]. In azuki bean [*V. angularis* var. angularis (Willd.) Ohwi and Ohashi]], QTL mapping was done for pollen viability trait under heat stress and identified two QTLs (i.e., *HQTL1* and *HQTL2* [135,136]. In pea, genetic bases of several traits including agronomic and seed quality traits [59,64], disease resistance [137,138], seed mineral concentrations [139], seed lipid content [140], salinity tolerance [141], and frost tolerance [142], have been uncovered through molecular mapping. Despite its importance, only limited studies have been carried out to identify genomic regions associated with stress tolerance in pea [143]. Therefore, more efforts are required to use the available genomic resources for mapping/tagging the genes/QTLs controlling the traits imparting in heat stress tolerance in cool season grain legumes.

### 3.2. Transcriptomics, Transcription Factors and Candidate Genes

Next generation sequencing (NGS) and RNA sequencing have led to unraveling and understanding many heat-tolerant candidate genes in different crops species [144,145,146,147]. Transcriptome analyses have been conducted in many legumes including cool season grain legumes for heat tolerance. For instance, in cowpea, the expression of various thermo-tolerant genes has been analyzed using cDNA–AFLP [148]. Efforts have been made to understand the genetic mechanism underlying heat shock factors (HSF) which play a vital role for survival under heat stress in many crop species. HSF-ESTs (expressed sequence tags), have been identified in *Lotus japonicas* (19), *Medicago truncatula* (21) and soybean (25) [149]. Transcript expression of *VfHsp17.9CII* gene in faba bean showed its increased accumulation and made 620-fold changes under high temperature [150]. Furthermore, in soybean HSP genes (*HSP 20*, *GmHsfA1*) and their role in thermo-tolerance have been evaluated [151,152,153]. Under different stress conditions, transcription factors (TF) are pivotal in modulating cellular responses and there by activating the transcription of target gene. These heat stress transcription factors (Hsfs) mediate activation of heat-responsive genes and chemical stressors through the signal transduction chain [154,155]. WRKY TFs have been identified as a major family of transcriptional regulators in other plant species, which form an integral part of cell signaling pathways and influence the stress tolerance [156]. Heat-stress elements (HSE: 50-AGAAnnTTCT-30) or palindromic binding motifs of promoters of heat stress (*hs*) genes are found conserved in eukaryotes [157]. *Arabidopsis thaliana* (HsfA2) is a typical representative of plant Hsfs having a modular structure [158]. Sequence comparisons has indicated that the combination of a C-terminal activator motif (AHA motif) with the consensus sequence FWxx(F/L)(F/I/L) to an adjacent nuclear export signal (NES) represent a signature domain for many plant class A Hsfs; this domain has enabled the identification of more than 90 unassigned new plant Hsfs and Hsf fragments in EST databases [159,160]. APETALA2/ethylene response factor (AP2/ERF) superfamily and heat-shock protein 90 (HSP90) family are another important stress responsive gene family [161]. These families not only regulate responses against various biotic and abiotic stresses in plants but also play an important role in various developmental processes.

In recent years, genome sequence availability of many cool season grain legumes like chickpea [45], lentil [162], and pea [163] has greatly helped in understanding the mechanism underlying stress tolerance. In chickpea, *DNAJ*, *HSP 70* and *HSP 91* genes have been reported by using Illumina/Solexa sequencing [164]. In addition, complete transcriptome analysis of heat-responsive genes in heat-sensitive genotypes (ICC 5912, ICC 4567, and ICC 10685) and heat-tolerant genotypes (ICC 15614, ICC 1356, and ICC 92944) has been also reported in chickpea [165]. Major gene families for heat stress like AP2/ERF gene family has also been reported in an earlier study [166]. One AP2 domain and three ERFs clustered with AP2 sequences have also been identified in chickpea [166]. Deokar et al. [167], also identified 16 AP2 and 120 putative ERF TFs in chickpea. This study strictly identified and characterized the AP2s through the presence of two AP2 domains [167]. Furthermore, transcription factors (TFs) for heat tolerance have been reported in chickpea [166,168]. Car-WRKY has been reported to be a multi-stress responsive transcription factor and plays a crucial role in stress signal transduction pathways [169]. Genome-wide analysis of a *WRKY TF* gene model has revealed the presence of 78 WRKY TFs evenly distributed across eight chromosomes in chickpea [4]. Furthermore, in chickpea seven genes including *ARP6* (actin-related protein), *PIE1* (photoperiod independent early flowering 1), two *SEF* (serrated leaf and early flowering), and three *H2AZs* (histone 2A variant-Z, a thermosensor in plants) having homology with chromatin remodeling complexes (SWR1) in *Arabidopsis* were identified and analyzed for their expression under heat stress [170]. Of the seven genes, *PIE1* was up-regulated during podding but down regulated at the seedling stage. Higher tissue-specific expression of *PIE1* and *SEF* genes was observed in root, flower, pod wall, and grain tissues than in shoots. During pod development, all three *H2AZ* genes might function as thermosensors, with greater downregulation within 15 min, 1 and 6 h of the heat stress treatment [170].

“HSP90” is an important gene family for heat tolerance in legumes. In cool season grain legumes, protein sequences have been examined for the presence of histidine kinase-like ATPases (HATPase c) and HSP90 motifs. In chickpea, five *HSP90* genes have been identified [166] and the proteins encoded by these genes ranged from 648 to 818 amino acids in length with isoelectric points ranging from 4.79 to 5.45 suggesting the conserved nature of HSP90 proteins across the different legumes. Expression pattern of *AP2/ERF* and *HSP90* in chickpea under heat stress and RNA-seq data generated from leaf, root and flower tissues at vegetative and reproductive stages revealed a unique set of *AP2/ERF* genes that expressed in different tissues [166]. In the same study, genes *Ca_01566*, *Ca_14133* and *Ca_22585* were down regulated, whereas *Ca_14133*, *Ca_02170*, *Ca_02170* and *Ca_23799* were up-regulated in the vegetative tissues of heat-tolerant and heat-sensitive genotypes. However, in reproductive tissues, *Ca_02170*, *Ca_08436*, *Ca_00673* and *Ca_08436* were up-regulated and *Ca_23799* and *Ca_22585* were down regulated. Expression results prompt the identification of probable tissue and stage-specific candidate genes, which can counteract the given stress condition. Furthermore, proteomics analysis has identified 482 heat-responsive proteins in the tolerant genotypes [171]. Besides, other proteins like pyrroline-5-carboxylate synthase (P5CS) acetyl-CoA carboxylase, ribulose-1,5-bisphosphate carboxylase/oxygenase (RuBisCO), ATP synthase, sucrose synthase, phenylalanine ammonia-lyase (PAL) 2, glycosyltransferase and late embryogenesis abundant (LEA) proteins have also showed strong association with heat tolerance in chickpea. Several crucial proteins were induced by heat exclusively in the heat-tolerant genotype. Accumulation of osmoprotectants, protected membrane transport, ribosome and secondary metabolite synthesis, activation of antioxidant and defense compounds, amino acid biosynthesis, and hormonal modulation identified using comparative proteome profiling and pathway analysis can be important mitigating strategies for heat tolerance in chickpea.

Functional genomics facilitates the elucidation of the important role of candidate genes for expression of tolerance against abiotic stress in crop plants [172,173,174]. Among cool season grain legumes, five HSP90 candidate genes (*Ca_25811*, *Ca_23016*, *Ca_09743*, *Ca_17680* and *Ca_25602*) have been identified in chickpea through RNA-sequencing analysis of leaf, flower and roots at different growth stages [166]. Mining of the candidate genes for heat tolerance revealed 236 genes in 2.28 Mb (44.6–46.9 Mb) region in CaLG05 and 550 genes in 6.50 Mb (7.85–14.35 Mb) in CaLG06 in chickpea. Functional categorization showed association of many genes with biological processes (168 genes in CaLG05 and 365 genes in CaLG06) in the two genomic regions [172]. Gene ontology classification revealed that these putative candidate genes (11 in CaLG05 and 14 in CaLG06) known to function, directly or indirectly, as heat-stress response genes in several plant species. Of the 25 candidate genes, five genes encoded protein like farnesylated protein 6 (AtFP6), ethylene-responsive transcription factor ERF114, ethylene-responsive transcription factor CRF4, F-box protein SKP2B and ethylene-responsive transcription factor RAP2-11. These genes played key roles in heat acclimation and growth of plants under severe heat-stress condition [172]. Additionally, the role of several heat stress responsive proteins such as *β*-galactosidase, glucanase, sucrose synthase, cystathionine gamma-synthase, 1-aminocyclopropane-1-carboxylic acid oxidase, abscisate *β*-glucosyltransferase, late embryogenesis abundant proteins imparting heat stress tolerance in chickpea has been deciphered [171]. A total of five candidate genes, namely *Ca_00060* (encoding membralin protein) underlying GA11 marker, *Ca_12498* (encoding ribosomal protein) underlying CESSR159 marker, *Ca_25724* (encoding transcription initiation factor TFIID) underlying NCPGR150, *Ca_17429* (encoding GDP-fucose protein O-fucosyl transferase) underlying NCPGR13 marker and *Ca_08534* (encoding pentatricopeptide repeat) underlying NCPGR202 were deciphered. Likewise, genes encoding various transcription factors (TFs) [170,172,175], ribosomal proteins [176], pentatricopeptide proteins [177,178], TIC, REF6, aspartic protease, cc-NBSLRR, RGA3 [175], and GDP-fucose protein [179] showed their contribution in various abiotic stress including HS tolerance in various crop.

In lentil, NGS-based transcriptome analysis provided opportunity to identify candidate genes expressed under biotic and abiotic stress conditions, including heat stress [2,180,181]. The transcriptome analysis of heat sensitive and tolerant genotypes led to the identification of candidate genes related to physiological and pollen phenotypes, cell wall, and secondary metabolism in lentil [181]. This study identified the genes for PDCB (plasmodesmata callose-binding protein 3), phosphatidylinositol/phosphatidylcholine transfer protein SFH13, CDP-diacylglycerol–glycerol-3-phosphate 3-phosphatidyltransferase 1 chloroplastic, probable GPAT2 (glycerol-3-phosphate acyltransferase 2), O-acyltransferase, and phosphatidylcholine diacylglycerol choline phosphotransferase. Those were up-regulated in tolerant genotype under heat stress. A gene encoding pyruvate phosphate dikinase identified under heat stress conditions has been found to be responsible for producing the phosphoenolpyruvate (PEP). This metabolite is an essential compound of shikimate pathway that is responsible for production of secondary metabolites involved in heat tolerance. These genes were involved in different pathways in cell wall formation and secondary metabolites production that were affected under heat stress [181]. In another study, 76 upregulated and 47 downregulated candidate genes have been identified at the late reproductive stage under heat-stress conditions and identified an important role of tryptophan biosynthesis under heat stress in lentil [58].

#### 3.2.1. microRNA

Plant miRNAs are a class of small (20–24 nucleotides) ncRNAs that regulate gene expression negatively by either degradation of mRNA or by inhibiting translation [182]. Evidences have revealed that miRNAs play crucial role in plant responses to heat stress. However, stress-associated regulatory networks that involve the role and activity of miRNAs are not clearly understood. It is further complicated to unravel such mechanism by the fact that several genes are regulated by one miRNA and some genes are regulated by multiple miRNAs. In different plant species, miRNA genes has been reported to enhance desired agronomic traits due to their tissue-specific, stress- or senescence-induced and constitutive overexpression [183,184,185,186]. Furthermore, endogenous and artificial target mimicry, artificial miRNA genes, Meganucleases, TALENs, ZNFs, CRISPR/Cpf1 or CRISPR/Cas13a systems, CRISPR/Cas9 and pri-miRNA or mature miRNA topical delivery have been shown to be useful for modulating miRNA accumulation.

miRNA genes are up or down regulated in response to biotic [187] and abiotic (reviewed by Ferdous et al. [188], Hackenberg et al. [189]) stresses in numerous species including soybean, sugarcane (*Saccharum officinarum* L.), rice, maize, wheat and tomato (*Solanum lycopersicum* L.). Studies on the expression or accumulation of these miRNAs have provided several lines of evidence to better understand the regulatory networks associated with defense mechanisms against different types of stresses. From these findings, several biotechnological tools have been applied for fine-tuning these networks and improving tolerance to stresses in important crops. Specific and well-studied example of miRNA involved to diverse abiotic stresses includes miR398, particularly for heat stress. In *Arabidopsis*, miR398 has four target genes (*CSD1, CSD2, Cox5b-1* and *CCS1*), which are highly conserved in land plants [190,191]. Among these, CSDs, which are closely related to copper/zinc superoxide dismutases, are important scavengers of reactive oxygen species (ROS) and CSD/CCS are involved in the negative regulation of accumulation of ROS [192]. They are also associated with heat shock protein (HSF) and heat shock factor (HSF) synthesis [193]. miR398 was shown to be rapidly induced in response to heat stress, accompanied by the down regulation of its target genes *CSD1*, *CSD2*, and *CCS* [194]. Transgenic plants expressing miR398-resistant versions of *CSD1*, *CSD2* or *CCS* showed hypersensitivity to heat stress, while the *csd1*, *csd2*, and *ccs* mutants were more tolerant to heat stress, with increased HSF and HSP levels [193,195]. In addition, miR398 and its target CSDs were also found in the heat stress responses of *Brassica rapa* and *Populus tomentosa* [149,196], indicating that the mi R398-CSD/CCS pathway is widely involved in the heat stress response in plants.

Many miRNAs have been utilized by direct cloning and sequencing in several legumes including *M. truncatula*, chickpea, common bean, peanut, soybean and lotus, [197,198]. It has been demonstrated that both conserved as well as novel miRNAs are present in these species that may help in regulation of legume species-specific cell processes [197]. A total of 1256 sequences that belong to 285 miRNA families have been reported from legumes in a publicly available miRNA database, miRBase (http://www.sanger.ac.uk/cgi-bin/Rfam/mirna/browse.pl, accessed on 19 March 2013). Several sets of novel species specific (legume) miRNAs have been reported, including novel (87) and conserved (42) miRNAs in soy bean [198,199,200]. In addition, excess of 100 novel miRNAs were identified in *M. truncatula* [201,202,203]. Further, 16 conserved and six stress responsive miRNA families have been identified in common bean [204]. Based on sequencing approach and computational predictions, a large number of miRNA gene families (482), miRNA precursors (1039) and mature miRNA (1114) sequences have been detected from soybean and related legume species [205]. Further, NGS technology has also been successfully used to systematically identify stress-associated miRNAs [206,207,208,209,210,211,212]. Recent studies in various plants species suggested that miRNAs play an important role in abiotic stress tolerance like drought, cold and salinity tolerance. These studies included conserved miRNAs such as miR164, miR169, miR171, miR396, miR398, miR399, miR408 and miR2118 [213,214]. However, few studies demonstrated role of miRNAs in heat stress response in cool season grain legumes. In the case of chickpeas, the role of miRNA for stress response has been examined. It has been demonstrated that overexpression of miR408 leads enhanced drought tolerance in chickpea through the regulation of copper accumulation. The miR408 overexpression results plantacyanin transcript repression to regulate DREB and other drought responsive genes [186]. This tool can largely aid in future breeding programmes for various biotic and abiotic stresses in legumes as in wheat 70 miRNA based SSR markers have been identified and validated on a set of 20 terminal heat-tolerant and heat-susceptible genotypes for developing heat tolerant cultivars through marker assisted selection [215].

#### 3.2.2. Signaling and Metabolic Pathways 

**Figure 3:**
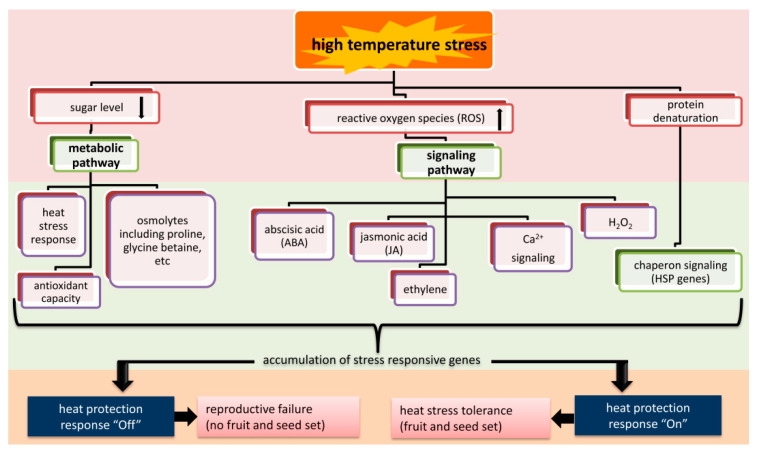
Flow-diagram showing how heat stress regulates reproductive development in legumes through sugar metabolic pathways and signalling pathway. The figures shows possible involvement of chain of chemicals/metabolites/signalling molecules in legume reproductive tolerance under heat stress.

Different signaling and metabolic pathways expressed in response of heat stress at different stages have been extensively studied and one of the schematic networks describing the mechanisms by which heat stress regulates reproductive development of legumes through sugar metabolism and signaling has presented in Figure 3.

In case of *Arabidopsis*, heat tolerance at seedling stage has been demonstrated. Mutants deficient in various pathways like hormone signaling including salicylic acid (SA), jasmonic acid (JA), abscisic acid (ABA), ROS systems and ROS regulatory systems and ethylene signaling as well as heat shock protein (HSP)-dependent pathways in the heat response have been analyzed [216]. *Arabidopsis* mutants deficient in S-nitrosoglutathione reductase (GSNOR), which metabolizes the nitric oxide (NO) adduct S-nitrosoglutathione, were more sensitive to heat stress compared with wild type [217], suggesting the involvement of GSNOR-dependent NO metabolism in heat tolerance of plants. Several heat sensors localized in different subcellular components have been identified. They include histone sensor in the nucleus, a calcium channel on the plasma membrane, and two unfolded protein sensors in the endoplasmic reticulum (ER) and the cytosol [218,219,220,221]. The deficiency in one of the heat sensors, cyclic nucleotide-gated channel 2 (CNGC2) resulted in enhanced heat tolerance in seedlings in addition to increased cytosolic Ca^2+^ level and enhanced accumulation of HSPs [222]. Furthermore, unfolded proteins in the ER and the cytosol have been identified to be linked with the heat sensing mechanism via ROS regulatory system [223]. (https://www.frontiersin.org/articles/10.3389/fpls.2018.00490/full, accessed on 4 December 2021)-B22Phytochrome B has been shown to be another heat sensor that mediates the switching of cellular status between growth-promoting mode and heat-acclimation mode [224,225]. Furthermore, involvement of key players of heat responses such as ROS regulatory systems, Ca^2+^ signaling, kinases and various hormones in defense responses has been demonstrated in previous studies [226,227,228]. However, cool season grain legume like chickpea and warm season grain legume like mungbean, increased H_2_O_2_ content and lipid peroxidation under heat stress were also observed [229]. Antioxidants, such as glutathione (GSH), ascorbic acid (AsA) and proline, play important roles in protecting plants from oxidative damage by scavenging ROS and thus enhance heat tolerance of legumes. Furthermore, there is often overproduction of different types of compatible organic solutes under abiotic stresses such as heat, drought, and salinity, among which proline and glycine betaine are important ones that act as osmoprotectants and ROS scavengers in stress tolerance of plants [230]. In chickpea, exogenous application of proline and glycine betaine improved the growth of seedlings under heat stress [231,232]. Proline may enhance heat tolerance of chickpea through alleviating the inhibition of heat stress on key enzymes in carbon and oxidative metabolism in seedlings [233]. Proline translocation also appeared to play an important role in controlling heat tolerance of reproductive development in cowpea [234]. Proline transporter genes have been identified among five heat-tolerant QTLs relevant to cowpea reproduction [134]. Therefore, it is speculated that proline and its transportation might regulate the response of legume reproduction under heat stress, which will be further testified by more direct evidence. However, little is known about the role of different signaling pathways, metabolites or metabolic pathways for controlling heat stress tolerance in lentil and other cool season grain legumes. Only few transcriptome studies identified the candidate genes that encoded synthesis of secondary and primary metabolites involved in heat tolerance in lentil [43]. However, metabolomics study can directly exhibit the metabolite changes induced by stress as compared with transcriptomics. Therefore, there is a need to apply metabolomics for exploring the metabolites involved in heat-stress regulation in cool season grain legumes similar to other crop plants [235,236]. Further, a combination of transcriptomics with metabolomics can help to elucidate the gene-to-metabolite pathways as investigated in rice in the response to heat stress [237].

## 4. Future Possibilities Genomic Intervention for Heat Stress Tolerance 

Knowledge of genomics enhanced in the past years can be used to intervene the development of heat stress tolerance in cool season grain legumes using the following ways.

### 4.1. Epigenetic Modifications

Environmental stresses including high temperature regulate the gene expression through DNA methylation, histone modification, miRNA expression modulation, alternate splicing etc. This is known as epigenetic modifications [238]. These changes help plants for better adaption under stress conditions [239] and provide thermotolerance [240]. It has been shown that priming of plants with mild or severe heat helps to maintain or acquire the heat stress memory, which increases themo-tolerance in subsequent exposure of heat-stressed primed plants [241,242]. The maintenance of thermotolerance is referred to as priming-mediated heat-stress memory [243]. The roles of epigenetic factors have been shown to control priming responses and to maintain the heat stress priming memory [244]. In *Arabidopsis*, alternative splicing has been identified an important molecular mechanism that underpin the heat shock priming-induced memory for enhanced heat stress tolerance [245]. This alternative splicing is regulated by epigenetic histone modifications [246]. Thus, transcriptional pattern of genes in this study has been observed under the control of epigenetics through alternate splicing and marked epigenetically hyper-inducted loci upon recurring exposure to heat stress. However, identification molecular mechanism involved in epigenetic control of heat stress tolerance is still challenging [247]. However, in lentil (a cool season grain legume), heat priming of the seeds for 6 h at 35 °C and a foliar treatment of γ-aminobutyric acid in combination helped to increase heat tolerance in sensitive genotypes by improving photosynthetic efficiency, chlorophyll concentration, and sucrose synthesis; and reducing the oxidative damage [248]. Epigenetic control of heat stress memory has also been identified through miRNA, which helps plants to adapt against heat stress through post-transcriptional regulators. Under heat stress, the expression of miRNAs and their targets are affected by DNA methylation [249]. This has been observed in several crop plants. For example, in poplar (*Populus simonii*), different heat-stress conditions methylated the miR393a, miR156i, miR167h, miR396e, and miR396g at CNG and CG sites [249,250]. However, efforts are needed to study the role of these in cool season grain legumes.

### 4.2. Mining Novel Allelic Variations for Heat Stress Tolerance

Genomics has made it possible to mine allelic variation for target traits. The roles of these genomic advancements have been discussed in cool season legumes for developing heat tolerance genotypes.

#### 4.2.1. Genome Editing

Genome sequences of several cool season grain legumes such as chickpea [45,251], pea [252], lathyrus [253], lentil [254], common bean [255] and adzuki bean [65,256], are available publically. These genomic resources have provided opportunity to generate allelic variation for genes controlling targeted traits using genome editing techniques. Generated allelic variation has opened up new breeding possibilities for mining the alleles for any given desirable trait [257]. The CRISPR/Cas9 genome editing approach has been used widely for modifying a genome in a targeted manner in many crops including rice, tomato, potato, cotton, soybean, maize, sorghum and wheat [257,258,259]. The genome editing produces novel genetic variability through engineering and repairing of pathways, and introduction of specific point mutations or insertions [260]. In the past years, efforts have been made to exploit the genome editing for enhancing heat stress tolerance by targeting genes for ethylene response and TFs in several crops [261,262,263,264]. In cool season grain legumes, genome editing of targeted genes has not been used for heat stress tolerance. However, in chickpeas, CRISPR/Cas9 editing was used for the *4-coumarate ligase* (*4CL*) and *Reveille* 7 (*RVE7*) genes, which were associated with drought tolerance leading to targeted mutagenesis [265]. In addition to this, CRISPR/Cas9-based genome editing has also used for a number of genes in other legume crops including model legume species [266].

#### 4.2.2. Targeting-Induced Local Lesions in Genome (TILLING)

Genomic-based TILLING (targeting induced local lesions in genome) approach has provided an opportunity to identify allelic variation for gene controlling a trait of interest [267]. For TILLING, a mutant population generated through ethyl methane sulfonate- (EMS), which produces point mutations distributed randomly in the genome, is required for identifying individuals with mutations in the target gene [268]. This has been used mainly for quality traits in several crops. One of the advantages is related with this approach that transgenic plants are not needed to generate a mutagenesis population. This approach has not been used to identify the allelic variation for a gene involved in g heat stress tolerance in cool season grain legumes. However, in other crops, TILLING approach has been used to screen mutagenesis population for mutations in the *HSP* genes. For example, new alleles for small *Hsp26* (*sHsp26*) genes were identified through TILLING that were found suitable for enhancing heat tolerance in wheat [269]. A mutated *HSP* gene enhanced heat tolerance in tomato has been identified using TILLING approach [270]. In common bean, TILLING population has been generated that can be used to screen mutation in the genes responsible heat stress tolerance [271]. 

### 4.3. Genomics of Underground Traits

Heat tolerance studies in cool season grain legumes have been focused mostly on above ground traits. However, in other crops, studies demonstrated that high temperature of soil highly affects the growth and development of plants than high air temperature under heat stress and thus roots also show their sensitivity to heat stress [272,273,274]. Thus, expression of pathways and proteins for thermo-tolerance in roots may be different than above-ground traits imparting in heat stress tolerance. Therefore, focuses have been given in other crops to studying the impact of heat stress on roots for enhancing knowledge of underlying mechanism and pathways involved in heat stress tolerance. Proteomics is a powerful approach to discover the proteins and pathways that are crucial for stress responsiveness and tolerance. Therefore, proteomic proofing has been done in *Agrostis* grass species that found up-regulation of sucrose synthase, glutathione–S–transferase and superoxide dismutase. This study also showed that heat shock protein Sti (stress-inducible protein) may contribute to the superior root thermotolerance and high phosphorylation of fructose-biphosphate aldolase under heat stress [272]. Proteomic knowledge in association with genomics helps to know the genes and pathways control the thermos-tolerance in roots under heat stress. Thus, a genome-wide transcriptional and proteomic profiling of root traits under heat stress in soybean showed differential expression of 1849 and 3091 genes in root hairs and stripped roots, respectively, in response to heat stress. This study identified 10 key regulatory modules controlling the majority of the transcriptional response to heat stress. Proteomic analysis in this study identified a variety of proteins changed their expression under heat stress and most of them showed their role in thermo-tolerance, chromatin remodelling and post-transcriptional regulation [274]. Further efforts are required for genomics and proteomic studies in cool season grain legumes.

### 4.4. Nanoparticles Based Genomics and Proteomics 

Nanotechnology is an emerging field for providing the new information on biological systems because interaction of nanoparticles with plants led to several morphological and physiological changes. Nanoparticles of minute sizes can penetrate in the cell organelles and nuclei leading to disruption of basic biological function and change in the structure and function of DNA [275,276]. Nanoparticles have both negative and positive effects on plants [277]. In a recent study, nanoparticles have been shown to have positive impact on plant by immunizing them against heat stress [278]. As nanoparticles made several changes in the profiling of proteins and transcriptomes, high-throughput transcriptomics, proteomics, and metabolomics approaches can help to understand these changes at molecular level in crop plants [279]. Thus, nanoproteomics and nanogenomics involving application of proteomics and genomics techniques aided by nanotechnology [280] may become more useful to understand biochemical and molecular responses crop plants for nanoparticles under heat stress. 

## 5. Concluding Remarks

During the last two decades, significant progress has been made on heat-stress tolerance and its component traits including number of filled pods per plot, total number of seeds per plot, % pod setting, chlorophyll concentration, photochemical reflectance index and canopy temperature, reproductive stem length, internode length and pod number in cool season grain legumes. Efforts have been made to develop the image-based techniques for high-throughput phenotyping of flowering intensity in cool season grain legumes [32]. In cool season grain legumes, significant progress has been made in development of genomic resources. However, the available information on genomics has not been fully utilized in breeding programmes. Limited efforts have been made to identify QTL/genes for heat stress tolerance. Moreover, all the identified genes have not been functionally validated. Therefore, sincere efforts are required to identify the functional and associated markers with heat stress tolerance in cool season grain legumes. These QTLs can be introgressed through marker aided conventional breeding in elite but heat sensitive grain legume cultivars. By the availability of knowledge about QTLs and markers, the use of molecular breeding to supplement conventional breeding will certainly give a new direction to legume breeding programmes. The availability of genome sequence for several cool season grain legumes has greatly helped in understanding the mechanism underlying stress tolerance in these crops. Further, it has become possible to achieve better resolution and improved understanding of genes expressed at transcriptome level under heat stress by applying NGS technology. It has been demonstrated that both conserved as well as novel miRNAs are present in legume crops like *M. truncatula*, chickpea, common bean, peanut, soybean and lotus that may help in regulation of legume species specific cell processes associated with heat shock protein (HSF) and heat shock factor (HSF) synthesis. The metabolomics study can directly exhibit the metabolite changes induced by stress as compared with transcriptomics. Only few transcriptome studies have identified the candidate genes that encode synthesis of secondary and primary metabolites involved in heat tolerance. Therefore, further progress in this direction needs to apply metabolomics for exploring the metabolites involved in heat-stress regulation in cool season grain legumes. In the future, genome editing and base editing using a variety of CRISPR/Cas9 systems can provide desirable mutants for traits imparting tolerance to heat stress in cool season grain legumes. Moreover, TILLING, genomics for underground traits, epigenetics and nanoparticle-based genomics and proteomics have opened up future possibilities for enhancing the heat-stress tolerance in cool season grain legumes.

## Figures and Tables

**Table 1:** Genomic resources in cool season grain legumes.

Crop	Reads/EST	Unigenes/Transcript	SSR	SNPs	References
Chickpea	-	160,883		1022	[44]
	-	2619	81,845	76,084	[45]
	-	53,409	4816		[46]
	-	34,760	4111	495	[47]
	-	103,215	26,252	26,082	[48]
	-	-	-	14,454	[49]
	-	37,265	4072	36,446	[50]
	-	43,389	5409	39,940	[51]
Lentil	1,380,000	25,592	-	-	[52]
	1,030,000	27,921	-	-	[53]
	119,855,798	20,009	-	-	[54]
	111,105,153	97,528	-	-	[55]
	58,621,121	77,346	-	-	[56]
	46,700,000	-	-	-	[57]
	26,165,023	96,824	-	-	[2,58]
	-	-	-	-	
Pea	1005.1 million		-	16,877	[59]
	-	-	-	10,739	[60]
		-	-	36,188	[61]
	18,552	10,086	586	-	[62]
	-	-	-	520	[63]
	-	-	-	340	[64]
	-	-	-	956	[61]
	3,042,418		-	35,455	[65]
			-	8822	[66]
	2,209,735	195,661	-		[67]
			-		
	40,903	10,506	-		[68]
			-	248,617	[69]
	432 million	27,145	-	-	[70]
	one billion reads	52,477	-	-	[71]
	69,706,469	48,628	-	-	[72]
	~55 million	81,774	-	-	[73]
	88 million	7946	-	-	[74]
	-	8899	3275	-	[75]
	-	10,800	2395	-	[76]
	720,324	70,682	2397	-	[77]
Grass pea	493,364		651,827	-	[78]
	570 million	27,431	3204	146,406	[79]
	46,994,629 + 72,566,465	134,914	200	4892	[80]
	399,648	14,386	-	-	[81]
Faba bean	-		-	14,552	[82]
	-	37,378	9071	-	[83]
	-		25,502 + 12,319	-	[84]
	1,247,881	343,325	-	560–2144	[85]
	87,269	-	-	39,060	[86]
	-	-	28,503	-	[87]
	304,680	60,440	802	-	[77]
Common bean		-	629	-	[88]
	3123	-	184	-	[89]
		-	-	7015	[90]
	418 million	-	-	346,819	[91]
	-	-	-	19,204	[92]
	-	-	-	17,190	[93]
	-	-	-	43,018	[94]
	-	-	-	12,697	[95]
	-	-	-	230	[96]
	21,026	7969	-	-	[97]
	-	3126	-	-	[98]
	-		-	1800	[99]
	7079	4219	-	-	[100]
	37,919	10,581	-	-	[101]
	9583	-	4764	-	[102]
	-	59,295	-	-	[103]
	900,000	30,491	-	-	[104]

**Table 2:** QTLs mapping for traits associated with heat stress tolerance in cool season grain legumes.

Crop	Traits	QTL Name/No. of MTAs	Population Size	PVE (%)	Reference
Chickpea	filled pods/plot	*qfpod02_5*	292	12.03	[113]
	total number of seeds/plot	*qts02_5*	292	10.00	[113]
	grain yield per plot	*Qgy02_5*	292	16.56	[113]
	% pod setting	*q%podset06_5*	292	13.30	[39,113]
	chlorophyll content	-	206	17.2	[114]
Lentil	seedling survival	*qHt_ss*	142	12.1	[115]
	pod set	*qHt_ps*	147	9.23	[115]
Field pea	chlorophyll concentration	6	135	7–13	[116]
	photochemical reflectance index	2	135	9	[116]
	canopy temperature	2	135	6	[116]
	reproductive stem length	6	135	4–6	[116]
	internode length	6	135	5–7	[116]
	pod number	9	135	7–10	[116]

## Data Availability

The study did not report any data.
